# Effect of stress, anxiety and depression on unstimulated salivary flow rate and xerostomia

**DOI:** 10.15171/joddd.2017.043

**Published:** 2017-12-13

**Authors:** Neda Gholami, Behrous Hosseini Sabzvari, Alireza Razzaghi, Shilan Salah

**Affiliations:** ^1^Department of Oral Medicine, School of Dentistry, Zanjan University of Medical Sciences, Zanjan, Iran; ^2^General Dental practitioner, Zanjan, Iran; ^3^Safety Promotion and Injury Prevention Research Center, Shahid Beheshty University of Medical Sciences, Tehran, Iran; ^4^Department of Oral Medicine, School of Dentistry, Zanjan University of Medical Sciences,. Zanjan, Iran

**Keywords:** Anxiety, depression, saliva, flow rate, stress disorder, xerostomia

## Abstract

***Background.*** Unstimulated salivary flow rate can be influenced by different factors. This study was undertaken to evaluate the effect of stress, anxiety and depression on unstimulated salivary flow rate in adults.

***Methods.*** A total of 247 adult subjects, randomly selected from patients referring to Zanjan Dental School, were included in this investigation. The study procedures consisted of collecting salivary samples (in 5 minutes), completing a form for feeling of xerostomia and completing Depression Anxiety Stress Scale (DASS) Questionnaire to assess the severity of stress, anxiety and depression. Based on the results, the patients were categorized in four groups: Low salivary flow rate plus xerostomia (group 1, n=60), normal salivary flow rate plus xerostomia (group 2, n=59), low salivary flow rate without xerostomia (group 3, n=60) and normal salivary flow rate without xerostomia (control group, n=68).

***Results.*** The frequencies of subjects with severe and major depression in groups 1, 2 and 3 were 31.4%, 11.7% and 8.5%, respectively, with 4.4% in the control group. The frequencies of subjects with severe stress in groups 1, 2 and 3 were 21.7%, 3.3% and 11.9%, respectively, with 1.5% in the control group. The frequencies of patients with severe anxiety in groups 1, 2 and 3 were 50%, 30% and 61.1%, respectively, with 4.4% in the control group. Stress, anxiety and depression exhibited a statistically significant relationship with unstimulated salivary flow rate and xerostomia (P<0.05).

***Conclusion.*** Stress, anxiety and depression can influence unstimulated salivary flow rate and lead to xerostomia.

## Introduction

The term stress refers to a series of events that lead to a reaction in the brain (perceived stress), activating the physiological fight-or-flight response in the body.^1^ Anxiety is also a generalized unpleasant and vague sensation of fear and concern with an unknown origin, which one can feel. It consists of uncertainty, helplessness and arousal physiologically.^2^ In depression, the patient always feels despair, sorrow and anxiety, and all these thoughts, feelings and behaviors are the symptoms of the condition.^3^

Saliva is a complex combination of major and minor salivary gland secretions,^4^ which acts as a cleanser in the oral cavity, contributing to chewing foods and facilitation of swallowing.^5^ The saliva has a buffering effect, leading to neutralization of acids in the oral cavity and protection of the teeth. Saliva also strengthens the mucosal barrier and has antimicrobial properties.^6^

Unstimulated salivary flow rate is defined as the volume of saliva secreted by major and minor salivary glands in a minute without any stimulation. The normal range of unstimulated and stimulated salivary flow rates are 0.3‒0.5 and 0.5‒0.7 mL/min, respectively.^5,7^ Amounts less than the above suggest salivary gland dysfunction. Sometimes an individual with normal salivary flow rate complains of dry mouth, also called xerostomia.^4^

The prevalence of diminished salivary gland secretions varies in the general population.^8^ In general, xerostomia exhibits greater prevalence in females compared to males.^9^ A 15-year study reported a decrease in salivary gland function from 15% at age 50 to 6% at age 65.^10^

Reduced salivation can lead to some side effects such as speech problems, chewing disorders, inflammation of the mucosa (mucositis), oral *Candida* infections and mucosal atrophy. It can increase accumulation of plaque and decrease the saliva buffering capacity.^4^

Secretion of saliva might be affected by several factors such as stress,^11^ anxiety and depression,^12^ age,^13^ previous treatment or cancer radiation therapy,^14^ medications^5^ and some other factors. Reduction of salivary flow may also be related to the absence of one or more of the major salivary glands, infectious or non-infectious sialoadenitis, salivary gland tumors (benign or malignant) or systemic diseases, which might exert direct effects on the secretion of saliva.^15-18^ Furthermore, various conditions such as dehydration, anorexia and bulimia and eating disorders such as nutritional deficiencies, which are able to cause metabolic changes, are associated with reduced salivary function.^4^

Among the above-mentioned different risk factors affecting saliva, stress, anxiety and depression have been taken into consideration by some researchers due to their relatively strong role and available treatments. However, sufficient evidence on the relationship between these factors and salivation is not available.^19^ This study evaluated the effect of stress, anxiety and depression on unstimulated salivary flow rate of adults in Zanjan, Iran.

## Methods

The Ethics Committee of Zanjan University of Medical Sciences University approved this study under the code ZUMS.REC.1392.210.

### Inclusion and exclusion criteria

A total of 247 patients referring to Zanjan Dental School were selected randomly and, after signing an informed consent form, were included in this study. Then a questionnaire on demographic data as well as dental and medical history was submitted to the participants.

Individuals under 18 years of age, those with a positive history of systemic disease and those taking any medications (at the time of the project or 6 months before) or smoking were excluded.

### Data collection

Data were collected in this study in two parts; the first part included measurement of salivary flow rate and xerostomia. There are several ways to collect whole saliva, including draining, aspiration, spitting and use of absorbent materials; usually the last two methods are used.^4^ For sampling saliva, patients were ordered to avoid eating, drinking, smoking or any kind of oral stimulation, such as brushing from 90 minutes before collecting salivary samples to avoid the effect of circadian cycle on salivary flow rate. Salivary samples were collected from 8 to 9 in the morning. Then the participants were requested to spit saliva in special containers for 5 minutes every 60 seconds. In order to measure saliva, 2-mL syringes were provided. Then to assess xerostomia (dry mouth feeling), the symptoms were recorded in a specially prepared form by asking some questions and then based on the collected data mentioned above, the patients were classified into 4 groups:

1) Unstimulated salivary flow rate <0.1 mL/min with xerostomia: 60 subjects
2) Unstimulated salivary flow rate >0.1 mL/min with xerostomia: 59 subjects
3) Unstimulated salivary flow rate <0.1 mL/min without xerostomia: 60 subjects
4) Unstimulated salivary flow rate >0.1 mL/min without xerostomia: 68 subjects

The second part consisted of evaluating depression, anxiety and stress by using the DASS questionnaire (Depression, Anxiety and Stress Scale),^20^ which is the standard questionnaire in this area. First the questionnaire was translated into Persian by two oral medicine specialists and the validity of its content was confirmed. Cronbach's alpha coefficient was used to assess the reliability and for each of the areas of depression, stress and anxiety the coefficients were 0.826, 0.797 and 0.744, respectively. The mean Cronbach's alpha coefficient for three areas was 0.798.

The questionnaire consists of 42 questions in 3 parts: depression, stress and anxiety. Each section has 14 questions that are coded from 0 to 3 (never = 0, a little = 1, sometimes = 2 and always = 3) and the score range of each part is 0‒42.^20^ After completing the questionnaire, depression, stress and anxiety scores for each individual were determined as normal, mild, moderate, severe or very severe according to the score ranges' reference (Table 1).

**Table 1:** Score ranges' reference used to determine depression, stress and anxiety levels based on DASS questionnaire ^20^

**Severity of psychological disorder**	**Depression**	**Stress**	**Anxiety**
**Normal**	0‒9	0‒14	0‒7
**Mild**	10‒13	15‒18	8‒9
**Moderate**	14‒20	19‒25	10‒14
**Severe**	21‒27	26‒33	15‒19
**Very severe**	28‏+	34‏+	20‏+

### Data analysis

In this study descriptive and analytical analyses were used. Descriptive analyses such as means and standard deviations were used to analyze quantitative data, and frequencies and percentages were used to analyze qualitative variables. In addition, for analytical purposes, chi-squared and Fisher’s exact tests were used. Statistical significance was defined at P<0.05. Statistical analyses were carried out with SPSS 18.

## Results

**Figure 1:**
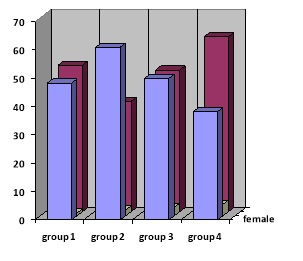
Demographic chart regarding gender distribution in four groups (frequency percentages).

Of a total of 247 patients in groups 1 (n=60), 2 (n=59), 3 (n=60) and 4 (n=68), respectively, 30, 23, 31 and 42 were male (Figure 1) and the average ages in groups 1 to 4 were 30.47±8.39, 31.68±11.41, 30.88±8.86 and 31.23‏±9.23, respectively. Statistical analysis showed no difference between the groups in demographic characteristics (P≥0.05).

Based on the results (Table 2), the frequency of subjects with severe and very severe depression score in the group exhibiting diminished salivary flow and xerostomia was 31.7%; in the group exhibiting diminished salivary flow without xerostomia the frequency was 11.7%; in the group with normal salivary flow and xerostomia the frequency was 8.5% and in the control group it was 4.4%.

**Table 2:** Comparison of depression, anxiety and stress between the four study groups

**Psychological disorder**	**Comparison of groups**		P-value
	j	i	
**Depression**	2	1	<0.01*
	3	1	<0.01*
	4	1	<0.001*
	3	2	<0.01**
	4	2	<0.001**
	4	3	<0.01**
**Stress**	2	1	<0.01*
	3	1	<0.01*
	4	1	<0.001*
	3	2	<0.01**
	4	2	<0.001**
	4	3	<0.01**
**Anxiety**	2	1	<0.001**
	3	1	<0.01*
	4	1	<0.001*
	3	2	<0.01*
	4	2	<0.001*
	4	2	<0.01*

‏*c^2^test ‏**Fisher’s exact test

The results also revealed that the frequency of subjects with severe and very severe stress scores in the group exhibiting diminished salivary flow and xerostomia was 21.7%; in the group exhibiting diminished salivary flow without xerostomia the frequency was 3.3%; in the group with normal salivary flow and xerostomia it was 11.9%, with 1.5% in the control group.

Analysis of data showed that the frequency of subjects with severe and very severe anxiety scores in the group exhibiting diminished salivary flow and xerostomia was 50%; in the group exhibiting diminished salivary flow without xerostomia it was 30%; in the group with normal salivary flow and xerostomia it was 61.1%, with 4.4% in the control group.

After one-by-one comparison of different groups, it was found that there was a significant relationship (P<0.01) between psychological disorders such as depression, stress, anxiety and xerostomia and unstimulated salivary flow rate.

## Discussion

Considering the different functions of saliva in the oral cavity, any change in its volume or composition might lead to malfunction.^4,5^ The results revealed that stress, depression and anxiety had a significant relationship with unstimulated salivary flow rate and xerostomia, consistent with the results of the majority of previous studies in this area but the innovation and importance of this research was to apply DASS questionnaire. By using this reliable, brief and comprehensive psychological scale three different major psychological disorders (stress, anxiety and depression) could be assessed simultaneously.^20^ Moreover, in this evaluation xerostomia (feeling of dry mouth) was considered in association with hyposalivation (reduction in the quantity of saliva) and the sample size was larger than that in some other studies.^5^

There are two techniques to evaluate the effect of mental disorders on saliva. A number of researchers have explored the effects of psychiatric medications on saliva^5^ and some studies, like this one, have examined the psychological disorders directly affecting salivation.

Although psychological processes that are independent of salivary secretion may be related to xerostomia, it is noteworthy that depression, by stimulation of anticholinergic mechanisms, can reduce salivary flow rate. Therefore, psychological conditions might affect both salivary flow rate and xerostomia. Furthermore, it was observed that salivary cortisol levels increased during stress, followed by changes in the composition of saliva.^5^

Borhan et al,^5^ in a study similar to the present study in relation to the groups but with smaller sample size, showed that stress and depression play a significant role in reducing the salivary flow rate and in increasing the incidence of xerostomia. In this regard, Bergdahl and Bergdahl^21^ evaluated 1202 individuals in three groups and similarly showed that unstimulated salivary flow rate under 0.1 mL/min and xerostomia are seen more frequently in patients with depression, anxiety and stress.

In relation to depression, Scarablot et al^22^ showed a significant relation between reduced salivary flow rates and depression and sleep disorders, consistent with our findings. This study also revealed that women with depression have more burning sensation and xerostomia than men.

Similarly, Hugo et al^23^ reported that stress can result in salivary gland hypo-function, which will reduce salivary flow. Matos Gomes et al^24^ applied Oral Health Questionnaire and Lipp Inventory of Stress Symptoms for Adults (ISSL)‏ and found a significant relationship between stress of exams and a decrease in salivary flow rate and total concentration of salivary proteins. Thirty-eight medical students were enrolled in their research, consistent with the results of this study and a study by Baharvand and Hemati.^9^ In the latter study, it was shown that 41.9% of subjects with oral dryness feeling had psychological disorders (depression, delirium*,* dementia and sleep disorders).

However, Naumova et al^25^ utilized the State-Trait Anxiety Inventory questionnaire (STAI) and reported different results in Sixty-four healthy male dental and medical students. Enzyme-linked immunosorbent assay was used to assess the physiological stress marker, cortisol. The researchers suggested other possible responses through salivary proteins that increase with the acute stress stimuli but acute stress (public talk) did not affect salivary flow rate. Such difference in the results might be attributed to various reasons, including the fact that salivary gland function might be influenced by age and gender.^3,26^ Noteworthy, Naumova et al excluded female subjects in order to avoid the effect of female hormone levels on cortisol measurements. Furthermore, different levels of stress and the duration of stress have different effects on immune function.^27,28^ Queiroz et al^27^ showed that acute stress (like exams) was capable of decreasing salivary levels but mild stress (like PMS [Premenstrual syndrome] ) had no effect on salivary flow rate. In the present study, “stress” as a general psychological disorder has been evaluated and because of wide range of data on features of stress, anxiety and depression we could not assess different types of stress (regarding the severity or acuity) in detail. This is the reason for the difference between the results of this study and the latter two investigations.

In another investigation Di Loreto^28^ evaluated the effect of stressful events in two groups (with and without anxiety). They found significant relationships between hyposalivation and rise of cortisol levels. Bergdahl and Bergdahl^21^ in their study observed a significant relationship between psychological factors (such as anxiety) and xerostomia or taste disorders. Both these researchers reported results similar to the present study.

Given the results of this study, we suggest further research with larger sample sizes and other standard questionnaires or investigations into the relationship between stress (acute or mild), anxiety and depression (each one individually); whole saliva or salivary flow rate also should be considered. Moreover, other factors affecting salivary flow rate such as age, gender, systemic disorders, medications, etc should be evaluated in separate investigations. Additionally, further research appears to be crucial to find other suitable criteria for evaluation of xerostomia.^29^

## Conclusion

Psychological variables such as anxiety, stress and depression have a significant effect on reducing salivary flow rate and on xerostomia.

## Acknowledgments

This article was derived from a thesis submitted by Behrous Hosseini Sabzvari for a doctorate degree in general dentistry in the School of Dentistry, Zanjan, Iran.

## Author’s contributions

NG was responsible for the concept or design of the work. BHS collected data for the work. AR interpreted data. SS drafted the work and revised it critically for important intellectual content. All the authors participated in the literature review

## Funding

This study was funded by Zanjan University of Medical Sciences.

## Competing interests

The authors declare no competing interests with regards to the authorship and/or publication of this article.

## Ethics approval

This project was approved by the Ethics Committee of Zanjan University of Medical Sciences (reference number ZUMS.REC.1392.210). Before starting the study, all the subjects signed an informed consent form.
